# Implementing stratified care for acute low back pain in primary care using the STarT Back instrument: a process evaluation within the context of a large pragmatic cluster randomized trial

**DOI:** 10.1186/s12891-020-03800-6

**Published:** 2020-11-25

**Authors:** Addie Middleton, G. Kelley Fitzgerald, Anthony Delitto, Robert B. Saper, Katherine Gergen Barnett, Joel Stevans

**Affiliations:** 1 New England Geriatric Research Education and Clinical Center, VA Boston Healthcare System, 150 South Huntington Avenue, Boston, MA 02130-4817 USA; 2 Department of Physical Therapy, University of Pittsburgh, Pittsburgh, PA USA; 3 School of Health and Rehabilitation Sciences, University of Pittsburgh, Pittsburgh, PA USA; 4 Department of Family Medicine, Boston Medical Center, Boston, MA USA; 5 Health Policy Institute, University of Pittsburgh, Pittsburgh, PA USA

**Keywords:** Low back pain, Risk stratified care, Primary care, Physical therapy

## Abstract

**Background:**

Although risk-stratifying patients with acute lower back pain is a promising approach for improving long-term outcomes, efforts to implement stratified care in the US healthcare system have had limited success. The objectives of this process evaluation were to 1) examine variation in two essential processes, risk stratification of patients with low back pain and referral of high-risk patients to psychologically informed physical therapy and 2) identify barriers and facilitators related to the risk stratification and referral processes.

**Methods:**

We used a sequential mixed methods study design to evaluate implementation of stratified care at 33 primary care clinics (17 intervention, 16 control) participating in a larger pragmatic trial. We used electronic health record data to calculate: 1) clinic-level risk stratification rates (proportion of patients with back pain seen in the clinic over the study period who completed risk stratification questionnaires), 2) rates of risk stratification across different points in the clinical workflow (front desk, rooming, and time with clinician), and 3) rates of referral of high-risk patients to psychologically informed physical therapy among intervention clinics. We purposively sampled 13 clinics for onsite observations, which occurred in month 24 of the 26-month study.

**Results:**

The overall risk stratification rate across the 33 clinics was 37.8% (range: 14.7–64.7%). Rates were highest when patients were identified as having back pain by front desk staff (overall: 91.9%, range: 80.6–100%). Rates decreased as the patient moved further into the visit (rooming, 29.3% [range: 0–83.3%]; and time with clinician, 11.3% [range: 0–49.3%]. The overall rate of referrals of high-risk patients to psychologically informed physical therapy across the 17 intervention clinics was 42.1% (range: 8.3–70.8%). Barriers included staffs’ knowledge and beliefs about the intervention, patients’ needs, technology issues, lack of physician engagement, and lack of time. Adaptability of the processes was a facilitator.

**Conclusions:**

Adherence to key stratified care processes varied across primary care clinics and across points in the workflow. The observed variation suggests room for improvement. Future research is needed to build on this work and more rigorously test strategies for implementing stratified care for patients with low back pain in the US healthcare system.

**Trial registration:**

Trial registration: ClinicalTrials.gov (NCT02647658). Registered January 6, 2016,

## Background

Improving outcomes for patients with low back pain is imperative. Low back pain is a prevalent condition, with over 25% of the United States’ (US) population experiencing back pain at some point over a three-month period [1, 2]. While the prognosis is generally considered good, recurrences are common (20–35%), and 5–20% of patients go on to develop chronic pain [3–5]. Low back pain is the second most common reason for healthcare visits, which drives a large proportion of patients into the primary care setting [2]. As a result, the economic impacts are substantial and include increased health care utilization and lost productivity (e.g., missed days of work) [2, 6]. Effective strategies for treating and managing this highly prevalent and costly condition are needed.

Stratifying patients with acute lower back pain based on risk of poor outcomes, such as developing chronic back pain-related disability, and matching treatment based on risk is an approach that shows promise for improving management of low back pain. For example, risk stratifying patients using the STarT Back risk-stratification tool produced superior patient outcomes and cost savings when compared to usual care in the United Kingdom (UK) [7, 8]. Based on these successes, the Matching Appropriate Treatments to Consumer Healthcare (MATCH) cluster randomized pragmatic trial adapted and implemented the stratified model of care shown to be effective in the UK into US primary care clinics [9]. The primary care teams and physical therapists working in the intervention clinics were trained on the stratified approach, and the STarT Back tool was integrated into the electronic health record (EHR) system in these clinics, along with information on the treatments recommended for low, medium, and high-risk patients with back pain [10]. Several implementation strategies were used to enhance uptake of the stratified approach, including changing records systems and work flow, developing and distributing educational materials, and offering centralized technical support [11]. Despite these efforts, no differences in disability were observed between patients with back pain treated in the intervention and control clinics at six months [9].

A process evaluation of the MATCH trial revealed that only 50% of patients with low back pain were stratified using the STarT Back Tool, and once stratified, providers did not increase their referrals to the treatments recommended for these patients based on their risk group [11]. This failure to fully implement the stratified approach may explain the lack of clinical impact. The trial in the UK that reported superior outcomes for patients receiving stratified care had much higher rates of referral (72%) to the treatments recommended for patients’ based on their risk group [8]. To be effective, stratified care requires more than just identification and stratification. The other essential component is provision of recommended treatments based on risk group.

The Targeted Interventions to Prevent Chronic Low Back Pain in High-Risk Patients (TARGET) trial also focused on improving outcomes for patients with low back pain through stratified care. TARGET was a multisite cluster randomized pragmatic trial designed to compare usual care to usual care plus referral to psychologically informed physical therapy for primary care patients with acute low back pain at high risk of transitioning to chronicity [12]. The trial was conducted in five geographically diverse health systems. The primary outcomes of interest were transition to chronic low back pain and low back pain-related disability at six months [12].

Both the TARGET and MATCH studies employed similar approaches to stratification; patients with low back pain could be identified and risk-stratified during primary care visits using the STarT Back tool, which was integrated into the EHR [12]. Both approaches included flexibility in who (e.g., front desk staff, rooming staff) identified patients with back pain and completed the risk stratification process, how (e.g., tablet, paper, verbal) risk stratification was completed, and the use of Best Practice Alerts to trigger a referral to recommended treatments [10]. In addition, the TARGET trial employed a number of additional strategies to improve identification, stratification, and utilization of recommended treatments. Bi-monthly audit and feedback reports, practice facilitation, and financial incentives for clinic staff were also provided to participating primary care clinics in the hopes of improving the risk stratification and referral processes.

The purpose of the present study was to complete a process evaluation ancillary to the TARGET Trial focused on the essential processes required for delivering stratified care, risk stratification and referral. The objectives were to: 1) examine variation in risk stratification and referral rates across primary care clinics and 2) identify barriers and facilitators related to implementation of these processes.

## Methods

The TARGET Trial was a multisite, cluster randomized, pragmatic trial conducted in five health systems, the University of Pittsburgh Medical Center (UPMC), Boston Medical Center, Intermountain Healthcare, John Hopkins University, and the Medical University of South Carolina. Recruitment began in May 2016 and ended in June 2018. The protocol for the trial has been published [12]. The trial adhered to CONSORT guidelines.

We used a sequential mixed methods study design to evaluate implementation at the 33 primary care clinics participating at the UPMC site. The trial was rolled out at UPMC as a quality improvement initiative, and 17 clinics were randomized to the intervention (usual care plus referral of acute high-risk patients to psychologically informed physical therapy) and 16 to the usual care control conditions. The population of interest in the TARGET Trial was adult primary care patients with evidence of acute low back pain management in the EHR [12]. Ethics approval was provided by the UPMC Quality Improvement Institutional Review Board (QI IRB 293 & 346).

For the process evaluation, we focused on two TARGET Trial process measures: 1) identification and risk stratification of all patients with back pain and 2) referral of high-risk patients to psychologically informed physical therapy. Per the study protocol, risk stratification was to occur at all participating clinics (*n* = 33) and referral of high-risk patients to psychologically informed physical therapy was to occur only at intervention clinics (*n* = 17).

### Identification and risk stratification

The first step of the risk stratification process was identifying patients with back pain, as few patients report back pain when scheduling their appointment. If not reported during scheduling, back pain could be identified by front desk staff, rooming staff, or clinicians. Front desk staff were responsible for checking patients into the clinic for their visit, while rooming staff typically escorted patients to treatment rooms, assessed vitals, and performed other tasks to prepare patients for their visit with the clinician. Clinicians evaluated, treated, and referred patients as appropriate. Once a patient was identified as having back pain by front desk staff, rooming staff, or a clinician, the individual could be risk-stratified to generate information to guide clinical decision-making.

The risk stratification process included two questionnaires. The first was the two-item Chronic Low Back Pain Questionnaire using the NIH Task Force on Research Standards definition for chronic back pain [12]. This was used to determine whether patients were in the acute or chronic stage of low back pain. If patients were in the chronic phase, no further screening was performed. If patients were in the acute phase, they then completed the STarT Back Screening Tool for stratification as low, medium, or high risk for developing persistent low back-related disabling symptoms [12, 13]. The risk stratification questionnaires could be completed on a study-specific tablet, which communicated with the EHR, or they could be completed verbally with responses entered into the EHR by clinic staff. Patients also had the option to complete the questionnaires through the healthcare system’s privacy protected patient portal during the seven days immediately preceding their office visit, if they reported back pain when scheduling their appointment. Once responses were in the EHR, the questionnaires were automatically scored, and the scores were available to all clinic staff and clinicians interacting with the patient. To ease the burden on clinicians, the risk-stratification process was designed to be completed by staff other than clinicians and could occur at different points in the workflow, such as check-in and rooming.

### Referral of acute high-risk patients to psychologically informed physical therapy

In the intervention clinics, Best Practice Alerts were automatically generated for patients with low back pain identified through risk stratification as acute high-risk and thus a candidate for psychologically informed physical therapy. The Best Practice Alert was configured to notify both clinic staff and clinicians. If a clinic staff member was acting on the Best Practice Alert, they could place an order for psychologically informed physical therapy in a “pended” status for review by the clinician, and the clinician could then choose to accept and sign the pended referral or to cancel or disregard it. If a clinician was acting on the Best Practice Alert, they could handle the whole process and generate a referral to psychologically informed physical therapy.

### Additional implementation strategies

Several additional implementation strategies were employed to facilitate the risk stratification and referral processes. Clinic staff and clinicians received in-person training on the processes prior to study initiation. Clinicians were given an online educational module on low back pain guideline-based care. Additionally, over the first two months of the study, a UPMC Quality Improvement (QI) staff member sent weekly email check-ins and offered regularly scheduled conference calls to address implementation challenges. Further clinician outreach was performed after study rollout. All clinicians were invited to one of three locations for an additional information session with the Principal Investigator and UPMC QI leadership. A meeting summary detailing issues raised by clinicians and solutions was sent to participating clinics following the completion of all three meetings. Remedial training was also provided to clinics upon request or as needed over the study period. Additionally, clinics were given a ‘Frequently Asked Questions’ guide and flyers to post in strategic areas of the clinic. The flyers encouraged patients to alert clinic staff if they were at the clinic to discuss back pain with their provider. To facilitate fit with clinic workflow, aspects of the risk stratification process were adaptable. Clinics could decide who performed the risk stratification process and when in the workflow it was completed.

Audit and feedback were provided in the form of bi-weekly clinic-level reports with risk stratification rates (proportion of patients with low back pain seen in the clinic over the specified time frame who completed the risk stratification questionnaires) and detailed information on the patients missed, including if and when the patient was scheduled to return to the clinic. A UPMC QI staff member was available to discuss these reports and clinic workflow by request. For the intervention clinics, the QI staff member also reviewed EHR reports daily and provided practice facilitation. The staff member would call or send an email to the clinic’s practice manager with information on patients who were identified as acute high-risk but had not had a referral to psychologically informed physical therapy pended in response to the Best Practice Alert. The practice manager was encouraged to work with clinic staff and clinicians to place an order for psychologically informed physical therapy, if clinically appropriate.

Incentives were provided to non-clinician staff (i.e., front desk and rooming staff) if clinics had rates of identification and risk stratification > 60% (all clinics) and rates of pended referrals to psychologically informed physical therapy > 80% (intervention only). The incentive was focused on “pended” referrals, rather than referrals, because it was targeted towards non-clinician staff, and their role was to pend the referral in response to the Best Practice Alert for clinician review. The incentive consisted of a small monetary bonus distributed among the clinic staff involved in the identification and referral processes. The bonus was paid twice at six-month intervals to clinics meeting the benchmarks and each clinic administrator had discretion over those staff eligible for the incentive based on the clinic’s workflow.

### Quantitative data sources and analyses

Data on risk stratification and referrals were pulled from the UPMC EHR by an honest broker. Deidentified data for patients who met eligibility criteria (i.e., a low back pain-related primary encounter diagnosis or order diagnosis and no contraindications for physical therapy) seen in participating UPMC clinics over the study period were provided for quantitative analyses.

We calculated risk stratification rates for all participating clinics. Rates were calculated as the proportion of patients with low back pain seen in the clinic over the study period who completed the risk stratification questionnaires. We calculated these rates for the overall cohort and each clinic individually. We also calculated risk stratification rates across points in the workflow (i.e., front desk, rooming, or clinician) for the overall cohort and each clinic individually. These workflow-specific rates were calculated using information in the EHR. Certain fields in the EHR are used by specific clinic staff. For example, front desk staff use the ‘Visit Type’ field and rooming staff use the ‘Encounter Reason’ field. Operationally these fields were used when a patient with low back pain was identified, and they allowed the staff member to launch the risk stratification questionnaires within the EHR. We calculated risk stratification rates based on which clinic staff identified the patient as having back pain (i.e., the field corresponding to the first occurrence of back pain in the EHR during the visit).

For intervention clinics, we calculated rates of referral of acute high-risk patients to psychologically informed physical therapy. Rates were calculated as the proportion of patients referred to psychologically informed physical therapy out of all patients seen in the clinic over the study period who were identified as acute high-risk. To examine the relationship between risk stratification and referral rates within clinics, we calculated a Pearson product-moment correlation coefficient.

### Qualitative data collection and analyses

We used the Consolidated Framework for Implementation Research (CFIR) as the overarching framework for our implementation evaluation. The CFIR is a comprehensive framework of constructs that may influence implementation [14]. The constructs are organized into the five domains: Characteristics of the Intervention, Outer Setting, Inner Setting, Characteristics of Individuals, and Implementation Processes [14]. We used the CFIR to develop our clinic observation guide, which included probes to elicit information of interest.

We performed onsite observations at purposively selected primary care clinics participating in the TARGET Trial. The goal was to include a sample that represented the full spectrum of performance on the risk stratification and referral processes. Clinics were selected based on performance, location, and availability. We scheduled onsite observations at three of the clinics in the top five for performance on the risk stratification process, three of the clinics in the bottom five for risk stratification, three of the clinics in the top five for referrals, three of the clinics in the bottom five for referrals, and two clinics not in the top or bottom five for risk stratification or referrals. One of the clinics was in the bottom five for both risk stratification and referrals, so the total number of clinics visited was 13. We weighted our sample towards clinics in the intervention group since these clinics were involved in both processes (risk stratification and referrals); however, we did want to include some clinics in the control group. Of the 13 clinics we selected, 10 were in the intervention group and three were in the control group. The clinic visits were completed in month 24 of the 26-month study period.

A UPMC QI staff member contacted clinics participating in the TARGET Trial and scheduled the onsite observations. AM and JS travelled to the sites and spent one to two hours observing clinic operations and interviewing clinic staff, which included primarily administrators, front desk staff, and rooming staff. Only two clinicians were available for observation or interview across the 13 clinics. Both investigators recorded field notes in real time and added reflective notes post-visit. The notes were imported into NVivo 12 (QSR International) software for analysis. All clinic visits were completed prior to analysis.

A descriptive content analysis approach was used to identify barriers to and facilitators of the risk stratification and referral processes. We started with an inductive approach. AM and JS collaboratively developed the initial codebook based on general impressions from the clinic visits and independent reviews of the field notes. The codebook was then refined iteratively; AM and JS independently coded two to three field notes, then compared and discussed coding and updated the codebook. Disputes were arbitrated by KF. Once a point was reached where no more changes were made to the codebook, the codebook was considered final and all field notes were re-analyzed using the finalized codebook. Coded text was then reviewed and organized into categories. Categories were classified as either a barrier, facilitator, or neutral factor. We then used a deductive approach and labeled text within the categories by CFIR domain and construct. We calculated the frequency of barriers and facilitators as the proportion of clinics where the factor emerged from the field note text.

## Results

### Quantitative analyses

#### Risk stratification

**Fig. 1:**
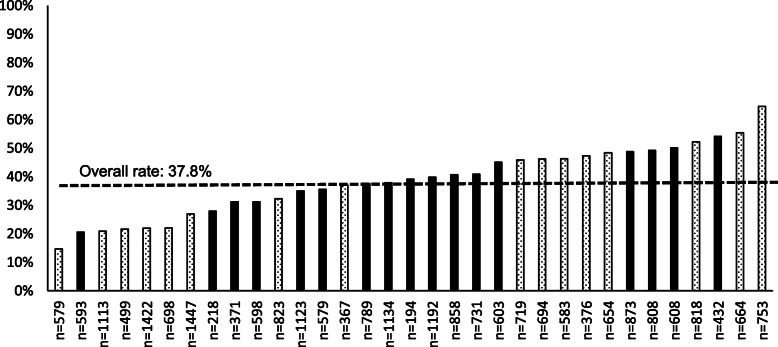
Risk stratification rates across the 33 primary care clinics participating in the TARGET Trial at the UPMC site. Note: “n” is the number of patients with low back pain seen in each clinic over the study period (i.e., the denominator of the rate). The dashed line represents the overall rate. Solid black bars represent clinics in the intervention group (*n* = 17) and patterned bars represent clinics in the control group (*n* = 16)

**Fig. 2:**
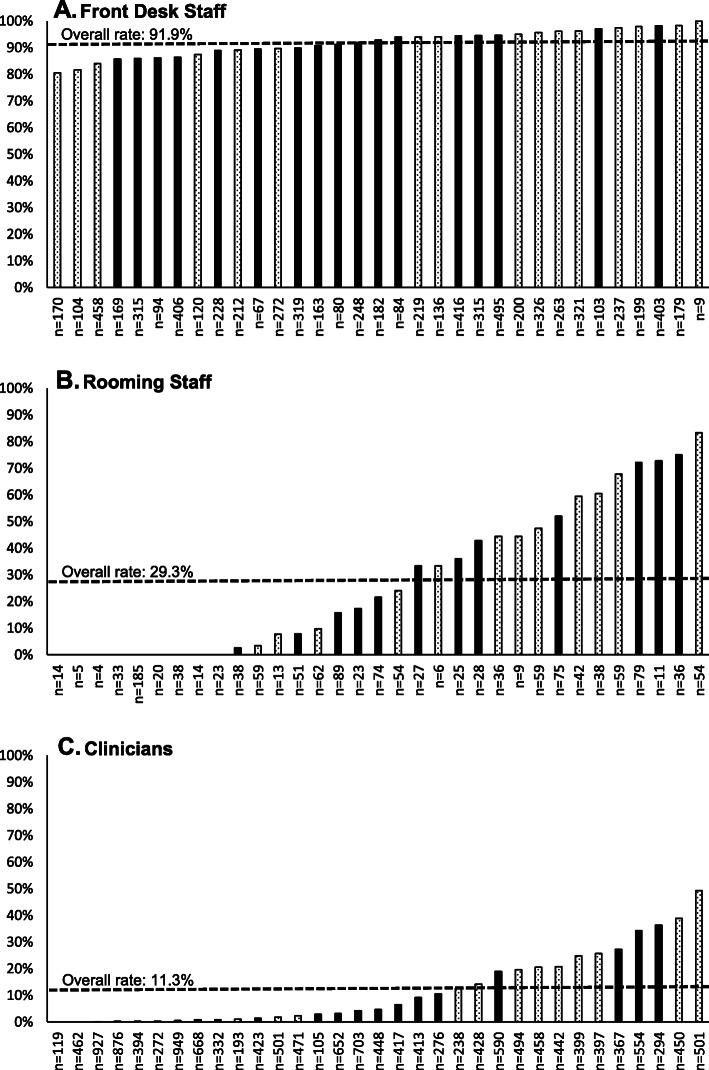
Risk stratification rates across points in the workflow (**a**. front desk staff, **b**. rooming staff, and **c**. clinicians) across the 33 primary care clinics participating in the TARGET Trial at the UPMC site. Note: ‘n’ is the number of patients identified as having low back pain at the indicated point in the workflow (i.e., the denominator of the rate). The dashed line represents the overall rate. Solid black bars represent clinics in the intervention group (*n* = 17) and patterned bars represent clinics in the control group (*n* = 16)

The overall risk stratification rate across the 33 primary care clinics participating in the TARGET Trial at the UPMC site was 37.8%, representing risk stratification of 9030 of the 23,913 patients with low back pain seen in the clinics over the study period. Risk stratification rates varied across clinics, ranging from 14.7 to 64.7% (Fig. 1). Risk stratification rates also varied across points in the workflow. Rates were highest when patients were identified as having low back pain by front desk staff (overall: 91.9%, range: 80.6 to 100%). Rates decreased as the patient moved deeper into the visit. The overall rate of risk stratification when low back pain was identified during the rooming process was 29.3% (range: 0 to 83.3%), and the overall rate was 11.3% (range: 0 to 49.3%) when low back pain was identified during the encounter with the clinician (Fig. 2).

#### Referral of high-risk patients to psychologically informed physical therapy

**Fig. 3:**
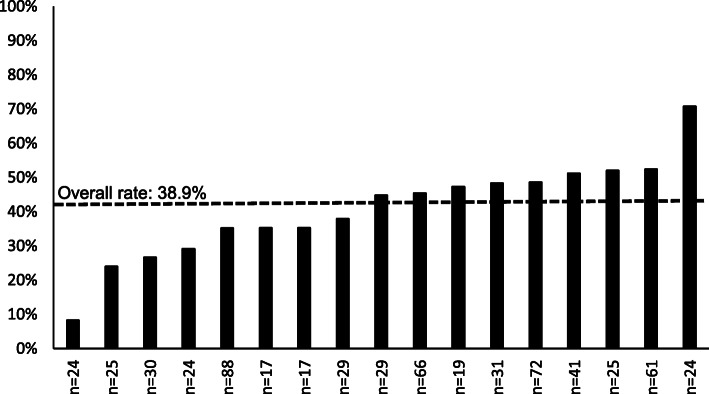
Rates of referral of acute high risk patients with low back pain to psychologically informed physical therapy across the 17 primary care clinics in the intervention group of the TARGET Trial at the UPMC site. Note: “n” is the number of patients with low back pain seen in each clinic over the study period (i.e., the denominator of the rate). The dashed line represents the overall rate

The overall rate of referral of acute high-risk patients with low back pain to psychologically informed physical therapy across the 17 clinics in the intervention group was 38.9% (range: 8.3 to 70.8%) (Fig. 3). The rate of referral of acute high-risk patients with low back pain to regular physical therapy in the control clinics was 30.2% over the study period. In intervention clinics, the rate of referral of acute high-risk patients to any form of physical therapy (psychologically informed physical therapy + regular physical therapy) was 59.2%. Risk stratification and referral rates were not correlated (r = − 0.15, *p*-value = 0.56) in the intervention clinics.

### Qualitative analyses

#### Barriers to risk stratification and referral processes

We used the qualitative data collected from our onsite observations and interviews with clinic staff to identify barriers to the risk stratification and referral processes. The barriers, corresponding CFIR domains/constructs, and illustrative quotes or examples are presented in Table 1. Difficulty identifying patients with low back pain was a barrier reported by 67% of the 13 primary care clinics visited. There were also barriers related to technology, with 79% of the clinics reporting issues with the use of tablets and 50% reporting issues with study activities in the EHR. Lack of physician engagement was a barrier reported by 46% of the clinics and competing priorities/lack of time was a barrier reported by 77% of the clinics.

**Table 1:** Barriers to the risk stratification and referral processes

Barrier	CFIR Domain - Construct	Illustrative Quote or Example
**Identification of Patients with Low Back Pain**	Characteristics of Individuals - Knowledge & Beliefs about the Intervention	*“The challenge was knowing the patient was here for back pain.”*
	Outer Setting - Patient Needs & Resources	*“Some patients are reluctant to talk about their issues. They want to wait until they’re talking to the physician.”*
**Technology**	Outer Setting - Patient Needs & Resources	*“Some patients get through the tablet and some just don’t, because of age or not tech-savvy.”*
	Process - Executing	*“I do have to go up to the front desk to get the tablet and the front desk has to change the visit type [in the EHR].”*
	Inner Setting - Networks & Communications	*Example: Some clinics had figured out solutions or work arounds to issues with study activities in the EHR while other clinics within their practice were not aware of these solutions/work arounds.*
**Physician Engagement**	Process - Engaging	*“Some [physicians] don’t even want the questionnaires done because they are busy, and they need to get in to see the patient.”*
**Competing Priorities**	Inner Setting - Relative Priority	*“We have a lot of #1 priorities and back pain is not one of them.”*

*Abbreviations*: *CFIR* Consolidated Framework for Implementation Research

#### Facilitators of risk stratification and referral processes

A facilitator of the risk stratification and referral processes was the compatibility with existing workflows. For example, chart reviews and schedule reviews were performed in some clinics to prepare for patients’ appointments and a few clinics had added checking for indications that the patient may have back pain to these existing processes. Other clinics asked all patients about pain as a standard part of the rooming process and had added probing questions specific to back pain to help identify patients appropriate for risk stratification. Over half (54%) of the clinics reported that the incentive was a facilitator, and almost all (93%) reported that they used the audit and feedback reports. However, how that information was used was variable. The adaptability of the risk stratification process was also a facilitator. Clinics were able to use the method that fit best in their setting. Thirty-one percent of the clinics had abandoned the tablets completely and used paper forms or verbally administered the risk stratification questionnaires, while 23% relied exclusively on tablets for risk stratification. Sixty two percent of the clinics used a mixed strategy and based the decision to use the tablet, paper forms, or verbally administer the questionnaires on the current situation and patient. Clinic staff sited challenges with keeping tablets charged and tablet/EHR communication issues as reasons for their abandonment. Note, the use of paper forms was contrary to the education provided to the clinics on the risk stratification process.

## Discussion

We report the results of a mixed methods, process evaluation examining implementation of a risk stratified approach to low back pain management in primary care clinics. While prior studies have focused on overall rates of risk stratification and referral [7, 9], we focused on variation in adherence to these processes. In our analysis of data from 33 primary care clinics participating at the UPMC site of the TARGET trial, we observed variation in rates of risk stratification and referral across clinics and across points in the workflow. Clinic staff’s knowledge and beliefs about the intervention, patients’ needs, technology issues, lack of physician engagement, and lack of time were all barriers to the risk stratification process. Adaptability of the process was a facilitator.

Complex interventions, such as the stratified approach, have multiple interacting components and are sensitive to local contextual factors [15]. As a result, successfully implementing complex interventions in primary care is challenging, and the best strategies for achieving behavior change in this environment are still unknown [16]. A recent systematic review found that most implementation strategies in primary care focus on change at the physician level (e.g., educational sessions) and are only modestly successful, while few strategies seek to make change at the organizational level [16]. The implementation strategy used in the TARGET trial was a combined approach but sought to emphasize change at the organizational level.

The implementation strategy used in TARGET was developed collaboratively with extensive engagement of physician, practice administration, and patient stakeholders. The ever-increasing demand on primary care physicians’ time and the numerous competing priorities they must juggle are well documented [17, 18]. Our stakeholders raised these issues repeatedly as barriers to initial adoption, as well as to implementation of the stratified approach. As a result, the TARGET implementation strategy was specifically designed using approaches shown to reduce burden on physicians [19]. The primary implementation strategy in TARGET was to shift responsibility for the identification and risk stratification of patients with low back pain to the clinic staff (i.e., front desk and medical assistants) and to leverage tablet technology for direct data entry and scoring within the EHR. Technology was further leveraged in the intervention clinics to generate Best Practice Alerts that notified medical assistants when a patient was eligible for referral to psychologically informed physical therapy (i.e., acute symptoms and high-risk). The goal of the TARGET implementation strategy was to minimize burden on physicians while facilitating risk stratification and referral of acute, high-risk patients with low back pain to appropriate treatment.

In our analysis of data from 33 of the primary care clinics participating in the TARGET trial, 38% of patients with back pain were successfully identified for risk stratification. In MATCH, approximately 50% of patients with low back pain seen in the intervention clinics were risk-stratified over the study period. However, we observed rates as high as 65% at the individual clinic level [9]. In our sample, the risk stratification rates varied across points in the workflow and were lowest when back pain was not identified until the patient was with the physician. While physicians were not expected to perform the risk stratification; it was expected that physicians would let other clinic staff know when back pain came up while they were in the room with the patient, so that the patient could be risk stratified and ultimately referred to appropriate treatments. MATCH’s educational approach placed a greater emphasis on physician training than TARGET, and this may partially explain differences in identification rates, as lack of physician engagement was frequently cited as a barrier in the TARGET study.

Overall, risk-stratification rates decreased as patients moved from the front desk, to the examination room, to the physician encounter. Primary care is a busy, fast-paced environment. Patients often have multiple health issues that need attention, and Medicare and other payors are holding providers accountable for quality metrics and instituting shared savings programs. As a result, clinic staff and clinicians have several tasks they need to complete with a patient during check-in, rooming, and the encounter. These competing priorities leave limited time for additional activities. Lack of time was cited very frequently as a barrier to risk stratification in each clinic visited, and these constraints intensified as the patient moved deeper into the clinic visit.

Based on these findings it is tempting to conclude that the risk stratification process should be conducted at the front desk; however, nearly all clinic staff reported difficulty identifying patients with low back pain. Interviewees reported that most patients were not comfortable talking to front desk or rooming staff about their health issues. Instead, they preferred to wait and talk to the “doctor”. This point should not be underestimated as patient reluctance to share information with staff has also been cited as a barrier to screening for depression in primary care [20]. Herein lies the paradox, it appears the front desk is the ideal place in the workflow to identify and risk stratify patients, but this is also the place where patients are the most reluctant to share information about their health concerns.

Several higher performing clinics developed solutions to overcome the barriers related to identification and risk stratification of patients with low back pain. These clinics incorporated patient identification and risk stratification into existing operational processes. For example, some clinics routinely conducted schedule reviews (front desk) or chart reviews (medical assistants) prior to the scheduled encounter. The reviews were performed to identify paperwork, tests, or services due to be completed at the upcoming encounter. This process is referred to as ‘scrubbing’ [21]. Clinics performing pre-visit scrubbing simply added checking for indications that the patient may have back pain to their existing processes. Other clinics routinely asked patients about pain as a standard part of the rooming process. These clinics added probing questions specific to back pain to help identify patients appropriate for risk stratification. Both approaches appeared to be effective; however, the strategy of choice may depend on contextual factors within individual clinics.

The TARGET implementation strategy sought to improve efficiency by leveraging technology. However, barriers related to technology were encountered. Clinic staff faced with multiple competing priorities did not have time to problem solve technology issues. While some clinics had developed alternative strategies or solutions, others had not, which impacted risk stratification rates. This inconsistency in solution identification highlights the need for effective strategies for spreading knowledge throughout organizations, such as Learning Collaboratives or Communities of Practice. Additionally, many clinics adapted the prescribed data collection procedures and opted to administer the STarT Back using paper forms. Clearly, technological alternatives do not efficiently integrate into all clinic workflows, highlighting the need for adaptability.

The TARGET trial devoted considerable implementation resources to ensure physical therapy referrals were made when appropriate within the intervention clinics. These strategies included 1) the use of Best Practice Alerts directed at both medical assistants and physicians, 2) allowing medical assistants to “pend” psychologically informed physical therapy orders for physician review, 3) audit and feedback reports, 4) practice facilitation, and 5) financial incentives for the clinic staff. Overall, the combined rate of referral of acute high-risk patients with back pain to physical therapy in the intervention clinics was double the referral rate in the control clinics. This result is promising considering that in the MATCH trial there was no impact on physicians’ referrals to recommended treatments; however, the higher referrals rates in TARGET must be viewed in light of the effort required to achieve them [9]. Further research is needed to better understand the barriers to clinician referral of appropriate patients to physical therapy.

Prior to initiating our process evaluation, we hypothesized that the primary care clinics participating at the UPMC site of the TARGET Trial would either have bought in to study activities and be fully engaged or would not be engaged at all. We expected clinics that were engaged to have high risk stratification and referral rates and clinics that were not engaged to have low rates of both. However, this was not the case. There was no association between risk stratification and referral rates. This disconnect appeared to be related to the division of study activities. Non-clinician staff were tasked with risk stratification, while physicians were responsible for referral. Interestingly, clinics were functionally divided into two business units. Staff reported to administrative leadership while physicians reported to clinical leadership. Little communication about risk stratification occurred between non-clinician and clinician staff. The lack of a team approach to stratified care may have been a limiting factor. Future efforts may need to promote a team approach with buy-in from the clinic as a whole.

### Limitations

We only evaluated risk stratification and referral process at one site of the TARGET trial. Findings may differ for other sites. During clinic visits, the staff were aware the study team was there to evaluate their processes for risk stratification of patients with low back pain and referrals to psychologically informed physical therapy. This may have influenced their responses and introduced bias, as staff may have provided responses that they thought were desirable. Another limitation was that we had minimal interaction with physicians during our clinic visits, and we were unable to fully explore their role in the risk stratification and referral processes.

## Conclusions

Adherence to key processes varied across primary care clinics participating in this pragmatic trial focused on improving outcomes for patients with low back pain through stratified care. Further exploration via on-site observations and interviews led to insights on barriers to and facilitators of the risk-stratification and referral processes. Clinic staff’s knowledge and beliefs about the intervention, patients’ needs, technology issues, lack of physician engagement, and lack of time were all barriers to the risk stratification process. Adaptability of the process was a facilitator. Future research is needed to build on this work and more rigorously test strategies for implementing stratified care for patients with low back pain in the US healthcare system.

## Data Availability

The datasets generated during and/or analyzed during the current study are available from the corresponding author on reasonable request.

## Abbreviations

US: United States
UK: United Kingdom
MATCH: Matching Appropriate Treatments to Consumer Healthcare trial
EHR: Electronic health record
TARGET: Targeted Interventions to Prevent Chronic Low Back Pain in High-Risk Patients
UPMC: University of Pittsburgh Medical Center
QI: Quality Improvement
CFIR: Consolidated Framework for Implementation Research
